# Reduced Androgen Receptor Expression Accelerates the Onset of ERBB2 Induced Breast Tumors in Female Mice

**DOI:** 10.1371/journal.pone.0060455

**Published:** 2013-04-08

**Authors:** Myles C. Hodgson, Garrett VanOstran, Sarah Alghamdi, Robert J. Poppiti, Alexander I. Agoulnik, Irina U. Agoulnik

**Affiliations:** 1 Department of Cellular Biology and Pharmacology, Herbert Wertheim College of Medicine, Florida International University, Miami, Florida, United States of America; 2 Department of Pathology, Mount Sinai Medical Center, Miami Beach, Florida, United States of America; 3 Department of Molecular and Human Genetics, Herbert Wertheim College of Medicine, Florida International University, Miami, Florida, United States of America; 4 Department of Obstetrics and Gynecology, Baylor College of Medicine, Houston, Texas, United States of America; 5 Department of Molecular and Cellular Biology, Baylor College of Medicine, Houston, Texas, United States of America; Baylor College of Medicine, United States of America

## Abstract

Androgen receptor (AR) is commonly expressed in both the epithelium of normal mammary glands and in breast cancers. AR expression in breast cancers is independent of estrogen receptor alpha (ERα) status and is frequently associated with overexpression of the ERBB2 oncogene. AR signaling effects on breast cancer progression may depend on ERα and ERBB2 status. Up to 30% of human breast cancers are driven by overactive ERBB2 signaling and it is not clear whether AR expression affects any steps of tumor progression in this cohort of patients. To test this, we generated mammary specific *Ar* depleted mice (MARKO) by combining the floxed allele of *Ar* with the MMTV-cre transgene on an MMTV-NeuNT background and compared them to littermate MMTV-NeuNT, *Ar^fl^/+* control females. Heterozygous MARKO females displayed reduced levels of AR in mammary glands with mosaic AR expression in ductal epithelium. The loss of AR dramatically accelerated the onset of MMTV-NeuNT tumors in female MARKO mice. In this report we show that accelerated MMTV-NeuNT-dependent tumorigenesis is due specifically to the loss of AR, as hormonal levels, estrogen and progesterone receptors expression, and MMTV-NeuNT expression were similar between MARKO and control groups. MMTV-NeuNT induced tumors in both cohorts displayed distinct loss of AR in addition to ERα, PR, and the pioneer factor FOXA1. *Erbb3* mRNA levels were significantly elevated in tumors in comparison to normal mammary glands. Thus the loss of AR in mouse mammary epithelium accelerates malignant transformation rather than the rate of tumorigenesis.

## Introduction

The majority of breast cancers originate in epithelial cells that line the ducts of the mammary glands. A number of alterations lead to the development of distinct tumor types from these cells. Two of the best described and therapeutically exploited drivers of transformation are estrogen receptor alpha (ERα) and v-erb-b2 erythroblastic leukemia viral oncogene homolog 2, neuro/glioblastoma derived oncogene homolog (avian), ERBB2, also known as human epidermal growth factor receptor 2 (HER2/Neu). However, additional markers are sought that denote molecular signatures that are better able to predict tumor progression, therapeutic response, and probability of recurrence.

According to different reports, 15 to 30% of breast cancers are driven by overexpression of ERBB2 [1], [2]. ERBB2 is part of a complex network comprised of four receptor tyrosine kinases (RTK), ERBB1-4. They can be bound by a variety of peptide hormones which cause receptors to homo- and/or heterodimerize and become active. ERBB2 does not have a ligand and is activated by partnering with itself or other family members [3]. Recent evidence suggests that the most critical partner for ERBB2 driven epithelial transformation and tumorigenesis is ERBB3. In human breast cancer cell lines with amplified ERBB2 expression, depletion of ERBB3 reduces cell proliferation to the same extent as depletion of ERBB2, while loss of ERBB1 (epidermal growth factor receptor (EGFR)) does not affect proliferation [4]. The activated form of ERBB3 was detected in human breast cancers with amplified ERBB2 expression [4]. Multiple mouse models overexpressing ERBB2 in mammary glands have been established, all of which lead to development of mammary tumors [5], [6], [7]. Similar to human breast cancer, ERBB3 plays a central role in murine breast cancer models. Ablation of endogenous *Erbb3* in mammary epithelial cells caused a decrease in ERBB2 induced tumor incidence from 93.3% to 6.7% [8]. In contrast, *Erbb4* ablation did not affect latency or histological grade of MMTV-Neu mouse mammary tumors [9]. As noted in human cancers, elevated expression of activated ERBB3 has been detected in transgenic mice overexpressing activated ERBB2 [10].

Estrogen, progesterone, and androgen receptors (ERα, PR and AR) are highly expressed in mammary epithelium and are essential for mammary gland development, function, and carcinogenesis. ERα inhibitors, such as tamoxifen, have been a mainstay of cancer prevention trials [11]. Aromatase inhibitors that block conversion of androgens to estrogens have been shown to protect against breast cancer progression in patients positive for ERα and PR [12], [13], [14]. Both ERα and PR are positive prognostic markers in breast cancer and their loss is associated with poor prognosis. Mouse models with either *Esr1* or *Pr* genes knocked out fail to develop functional mammary glands [15], [16] and both receptors are required for mouse mammary tumorigenesis [17], [18].

AR is expressed in the epithelium of the normal mammary gland and in approximately 70–90% of invasive breast cancers, a frequency comparable to that of ERα (70–80%) or PR (50–70%) [19]. In both normal and malignant human breast tissue there are cells that are positive for AR and ERα and cells that only express AR [20]. The role of AR in breast cancer is currently unclear and seems to depend on the cellular milieu. The expression of AR in ERα positive tumors is associated with less aggressive tumors and a more favorable prognosis [21]. However, AR expression in ERα negative and triple negative tumors is associated with poor prognosis and increased mortality [21]. In ERα negative breast cancer, ERBB2 overexpression significantly correlates with high AR and ERBB3 expression [22], [23], [24]. In MDA-MB-453 breast cancer cells that do not express ERα, AR activates ERBB2 signaling by direct androgen-dependent induction of WNT7B and ERBB3 expression [22]. In mouse models, stimulation of AR signaling mid-puberty suppressed epithelial proliferation and development of ductal extensions [25]. Global AR knockout significantly increased the susceptibility of the mammary gland to the chemical carcinogen, DMBA, presumably by elevating estrogenic activity [26].

Since AR is frequently expressed in ERBB2 amplified tumors, we asked how the loss of AR signaling would affect tumor incidence and progression in this cohort. We described here analysis of breast tumorigenesis in mice with overexpression of an activated form of ERBB2 and conditional deletion of the *Ar* allele specifically in the mammary gland epithelium (MARKO). Remarkably, the first tumors in female MARKO mice were detected almost 100 days earlier than in control mice. Similar to human ERBB2 driven breast cancer, tumors in ERBB2 transgenic animals show simultaneous increase in endogenous ERBB3 but not ERBB4 expression [8], [22], [27], [28]. We observed that tumors in both control and MARKO mice showed reduced gene expression of the steroid receptors *Er*α, *Pr*, *Ar* and their pioneer factor *Foxa1*. Our data suggest that ERBB2/ERBB3 driven tumorigenesis is opposed by AR and its loss results in earlier tumor development.

## Methods

### Animal Breeding and Genotyping

**Figure 1:**
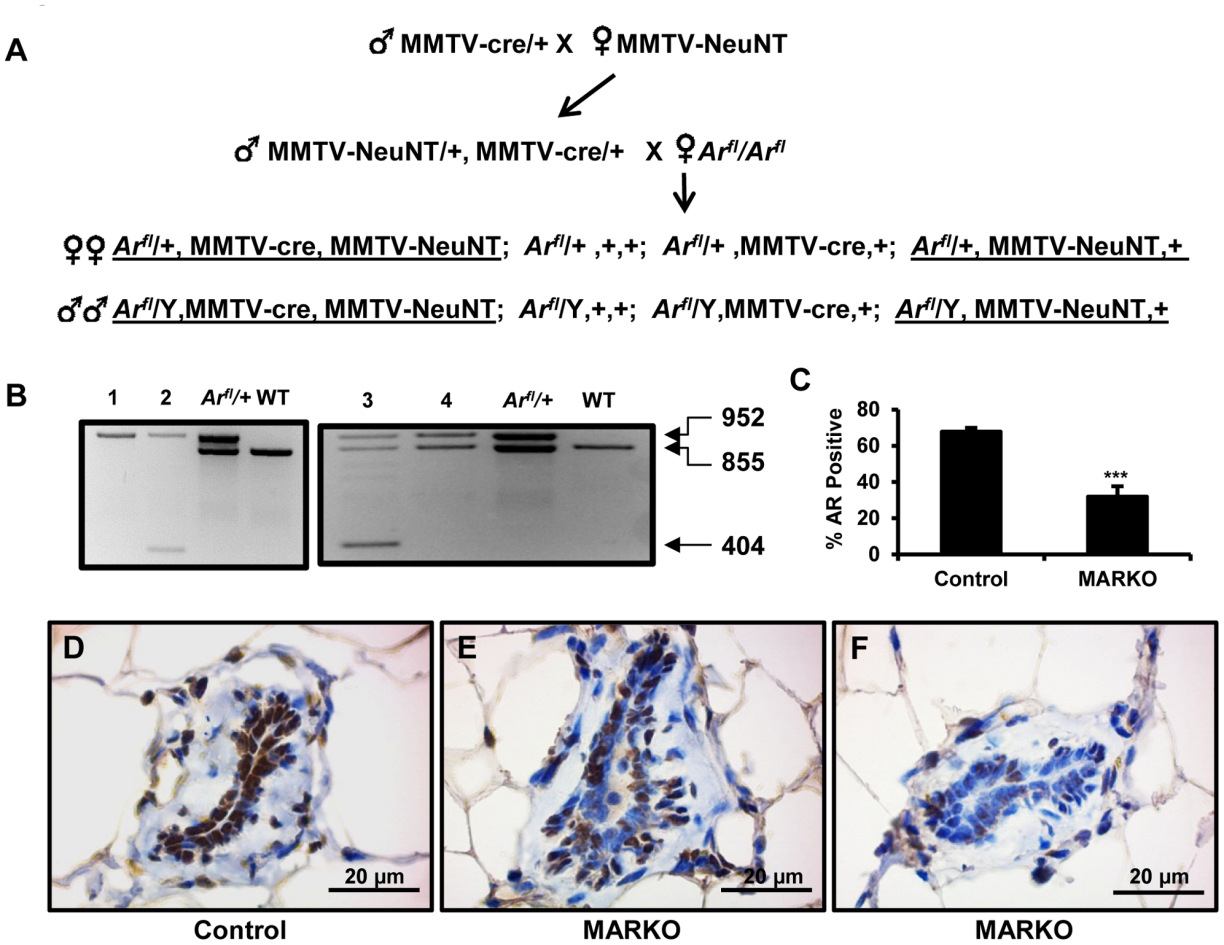
Characterization of mice with conditional knockout of AR in mammary glands (MARKO). A. Breeding strategy used to create experimental cohorts. Two generation breeding was used to produce tumor-prone MMTV-NeuNT (activated rat ERBB2) transgenic MARKO (male and females underlined on the left side) and Control (underlined on the right) mice. MARKO mice are positive for MMTV-cre transgene, specifically expressed in mammary glands. B. Recombination events in the genomic DNA from mammary ductal tissue dissociated from fat cells. Top band (952 bp) is derived from the non-recombined floxed *Ar^fl^* allele, middle band (855 bp) is from the wild-type *Ar^+^* allele, and the lower band (404 bp) is an amplicon derived from the *Ar^Δ^* allele, resulting from Cre/LoxP induced deletion of exon 2. *Ar^fl^/+* is a positive control for the floxed allele and WT is wild-type (*Ar^+^/Ar^+^*). Left panel is recombination in male mice and the right panel is recombination in female mice. No recombination is observed in mice lacking MMTV-cre (lanes 1 and 4), the deleted allele is present in mice with MMTV-cre (lanes 2 and 3) C. Reduced number of AR positive luminal epithelial cells in MARKO mammary glands. AR positive luminal epithelial cells in the mammary glands of Control (n = 5) and MARKO (n = 5) mice were counted and determined as a percentage of the total number of cells per gland (p = 0.0019). An average of 260 cells were counted per individual mouse. Immunohistochemical staining for AR expression in Control (D) and MARKO (E and F) mammary glands. Scale bar = 20 µm.

Animal studies were conducted in accordance with the humane standards of animal care, as outlined in the US National Institutes of Health Guide for the Care and Use of Laboratory Animals and all procedures were approved by the Florida International University Institutional Animal Care and Use Committee. All mice were maintained in a temperature controlled room, with 12-h light, 12-h dark photocycle and fed Teklad global 18% protein rodent diet chow (Harlan, Indianapolis, IN) and fresh water *ad libitum*. Transgenic mice (FVB-Tg(MMTV-ErbB2)NK1Mul/J) containing the activated rat *ERBB2* oncogene targeted to the mammary epithelium by the MMTV-LTR promoter (hereafter referred to as MMTV-NeuNT) [7] and mice with the Cre recombinase transgene under the control of the MMTV-LTR promoter (Tg(MMTV-cre)1Mam/J, MMTV-cre) [29] were purchased from Jackson Laboratories (Bar Harbor, ME, USA). Mice with floxed exon 2 of the X chromosomal androgen receptor gene (*Ar^fl^*) were described previously [30]. Deletion of Exon 2 causes a frame shift and production of an unstable amino-terminal fragment, completely lacking the DNA binding domain. Transgenic mice were genotyped by PCR from genomic DNA isolated by ear punches with primers specific to the *ERBB2* and *cre* transgenes and wild-type, floxed and deleted (*Ar^Δ^*) *Ar* alleles as described in the original publications. MMTV-cre, MMTV-NeuNT, *Ar^fl^/+* females and MMTV-cre, MMTV-NeuNT, *Ar^fl^/*Y males (MARKO, mammary gland specific AR knockout) and control MMTV-NeuNT, *Ar^fl^/+* females and MMTV-NeuNT, *Ar^fl^/*Y males were produced according to the breeding scheme shown in Figure 1.

### Incidence and Survival Study

MARKO females (n = 19) and males (n = 33) and littermate controls (females, n = 29; males, n = 31) were used in the survival study. All females were kept as virgins for the entire period of study. Mice were palpated at least twice a week to detect tumors. Tumor bearing mice were kept until they met euthanasia criteria which included tumor burden of 10% of the body weight or more, significant loss of weight, visible signs of distress, huddled posture, immobility, moribund appearance. The age of mice when a tumor was first detected (incidence) and the age of mice when euthanized due to tumor burden (survival) were recorded. At the time of euthanasia serum was collected, tumors and mammary glands were removed for histological analysis, RNA and DNA isolated, and other organs were examined for metastases. All female mice were sacrificed at 400 days of age and at 450 days for males. Kaplan-Meier survival plots were generated and compared by Log-rank test statistical analyses using GraphPad Prism software (GraphPad Software, San Diego, CA). P<0.05 was considered statistically significant.

### Histology and Immunohistochemistry

Mammary glands and tumors were fixed in 4% paraformaldehyde at 4°C overnight. Deparaffinized 5 µm sections of both tumors and normal mammary glands were immunostained for ERα, AR, ERBB2, and Ki67. All washes were done in TBS with 0.05% Tween-20. Antigen retrieval for ERBB2 was achieved by boiling for one minute, followed by fifteen minutes at 90°C in 1 mM EDTA pH 8.0. ERα and AR antigen retrieval was done by heating to 99°C in pH 6.0 10 mM sodium citrate buffer for fifteen minutes, with the addition of 1 mM EDTA for AR. Endogenous peroxidase activity was blocked using 1% H_2_O_2_ in methanol for ten minutes. Endogenous biotin was blocked using the Avidin/Biotin Blocking Kit (Vector Laboratories, Burlingame, CA), followed by blocking with 10% normal goat serum in TBS for 45 minutes. Immunostaining for AR (*RB-1358*, 1∶50, *Neomarkers*, Fremont, CA), ERα (SC-542, 1∶250, *Santa Cruz* Biotechnology, Inc., *Santa Cruz*, CA), and ERBB2 (mAB#4290, 1∶100, Cell Signaling Technology, Danvers, MA) were performed overnight at 4°C diluted in 3% BSA in TBS. Ki67 sections were incubated in primary antibody (RB-1510-P1, 1∶100, Neomarkers) in TBS for one hour at room temperature. AR, ERα, and ERBB2 stained sections were incubated with biotinylated goat anti-rabbit secondary antibody for 30 min at room temperature and then with streptavidin conjugated peroxidase for 30 min at room temperature. Ki67 was labeled using the Vectastain ABC kit (Vector Laboratories, Burlingame, CA). Staining was developed for all proteins using the ImmPACT DAB Peroxidase Substrate Kit (Vector Laboratories) and all slides were counterstained with haematoxylin.

Ki67 staining was quantified by counting the number of Ki67 stained cells in random fields of a total of >300 cells. Final counts were expressed as a percentage of cells positive for Ki67. AR staining was quantified by counting the number of positively stained luminal epithelial cells from randomly selected ducts. AR staining was determined as a percentage of the total number of luminal epithelial cells.

### Tissue Preparation and Gene Expression Analysis

Largest cellular compartment of the mouse mammary gland is fat. To remove fat cells, excised mammary glands were incubated in 1% w/v collagenase (Roche, Mannheim, Germany) dissolved in Earle’s Balanced Salt Solution at 37°C with constant agitation for 1 hour. Dissociated tissue was centrifuged at 1000 rpm for 5 min and upper layer of fat cells and overlaying buffer removed. An aliquot of pelleted cells was taken for DNA extraction using DNeasy Blood & Tissue Kit (Qiagen, Valencia, CA) and the remaining cells were resuspended in TRIzol reagent (Life Technologies, Grand Island, NY). RNA was extracted according to the manufacturer’s instructions. Five micrograms of total RNA was reverse transcribed to cDNA using the GoScript cDNA synthesis kit (Promega, Madison, WI). Quantitative PCR analysis of gene expression was performed using Roche Universal ProbeLibrary assays on a Roche 480 LightCycler. For a complete list of primers see Table S1.

### Hormone Analysis

Mice were anesthetized by isoflurane inhalation (Webster Veterinary, Devens, MA), and blood collected by ocular orbital bleeding. Serum was isolated by centrifugation and stored at –20°C until analysis. Testosterone and estradiol measurements were performed by radioimmunoassay (RIA) and ELISA (Calbiotech, San Diego, CA) respectively in the University of Virginia Center for Research in Reproduction Ligand Assay and Analysis Core (University of Virginia, Charlottesville). Sensitivity of the testosterone RIA assay was 10 ng/dL. Sensitivity of the estradiol ELISA assay was 7 pg/ml.

### Statistical Analysis

Student *t-*test for two groups and one-way ANOVA for multiple group comparisons were used to assess significance of differences. Differences were expressed as mean ±SEM; P<0.05 was considered significant. Tumor incidence data was analyzed using Gehan-Breslow-Wilcoxon test. All analyses were performed using the GraphPad Software package.

## Results

### Female MARKO Mice Display a Mosaic Pattern of AR Expression in the Mammary Gland

To assess the role of the AR in ERBB2 driven tumorigenesis, we crossed MMTV-cre, MMTV-NeuNT mice with *Ar^fl/fl^* mice to obtain MMTV-cre, MMTV-NeuNT, *Ar^fl^*/+ (or *Ar^fl^*/^Y^ males) MARKO mice and MMTV-NeuNT, *Ar^fl^*/+ female (or *Ar^fl^*/^Y^ males) control littermates (Figure 1A). The generation of homozygous *Ar* ^Δ^ female mice was not possible under the current breeding strategy, since MMTV-cre, MMTV-NeuNT, *Ar^fl^/*Y males, required for this breeding (Figure 1A), were infertile and displayed an under-masculinized phenotype due to *Ar* ablation in several male reproductive organs. This phenotype is similar to that observed for other ARKO models [30], [31], [32]. MMTV-Cre induced recombination of the *Ar^fl^* allele resulting in deletion of exon 2 was confirmed by PCR analysis of MARKO genomic DNA isolated from partially dissociated mammary glands (Figure 1B). The deleted allele was detected in all *Ar^fl^/+* females and *Ar^fl^/Y* males with the MMTV-Cre transgene. Due to X chromosome inactivation in females, a single allele of *Ar*, either control or exon 2– deleted, is expressed in each individual cell. This means that MARKO mammary epithelium is a mix of cells expressing normal levels of *Ar* and cells with no *Ar* expression. We counted AR positive luminal epithelial cells (Figure 1C) and found that the percentage of cells positively stained for AR in MARCO (∼32%) mammary glands was significantly lower than in controls (∼68%) (p = 0.0019). AR nuclear staining was detected in the mammary glands of both control and MARKO female mice. Nuclear AR staining was observed in the majority of luminal epithelial cells in the mammary glands of control females (Figure 1C and D). Varying frequency of AR expression was observed in MARKO (Figure 1C, E and F) mice, which showed a reduced percentage of positive AR nuclear staining in luminal epithelial cells. AR staining was observed in approximately 32% of cells. AR staining in adipose tissue was unaffected in all MMTV-cre mice.

**Figure 2:**
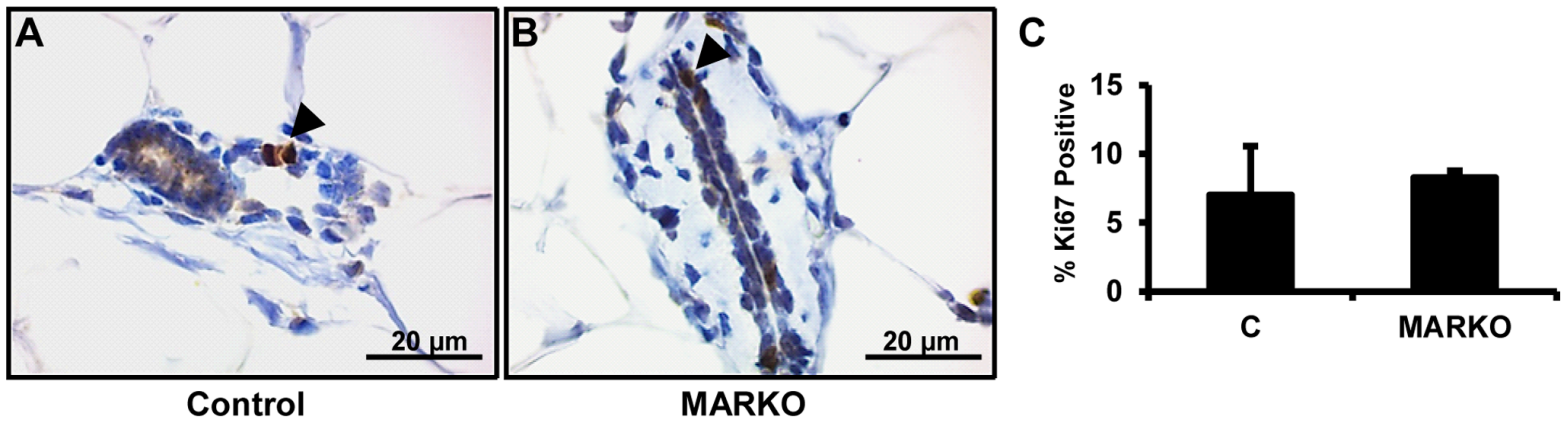
Control and MARKO mice show similar proliferation in the normal mammary gland. Representative pictures of normal Control (A) (n = 4) and MARKO (B) (n = 4) mammary glands stained for Ki67. Arrowheads denote Ki67 positive cells. C. Ki67 staining was quantified and is shown as the percentage of total epithelial cells positive and negative for Ki67 staining. Scale bar = 20 µm.

Global knockout of *Ar* in female mice was previously shown to increase luminal epithelial cell proliferation [26]. In our conditional knockout model, we observed similar luminal epithelial cell proliferation in female MARKO and control mice (Figure 2).

### Diminished AR Expression Promotes an Early Onset of ERBB2 Tumors

**Figure 3:**
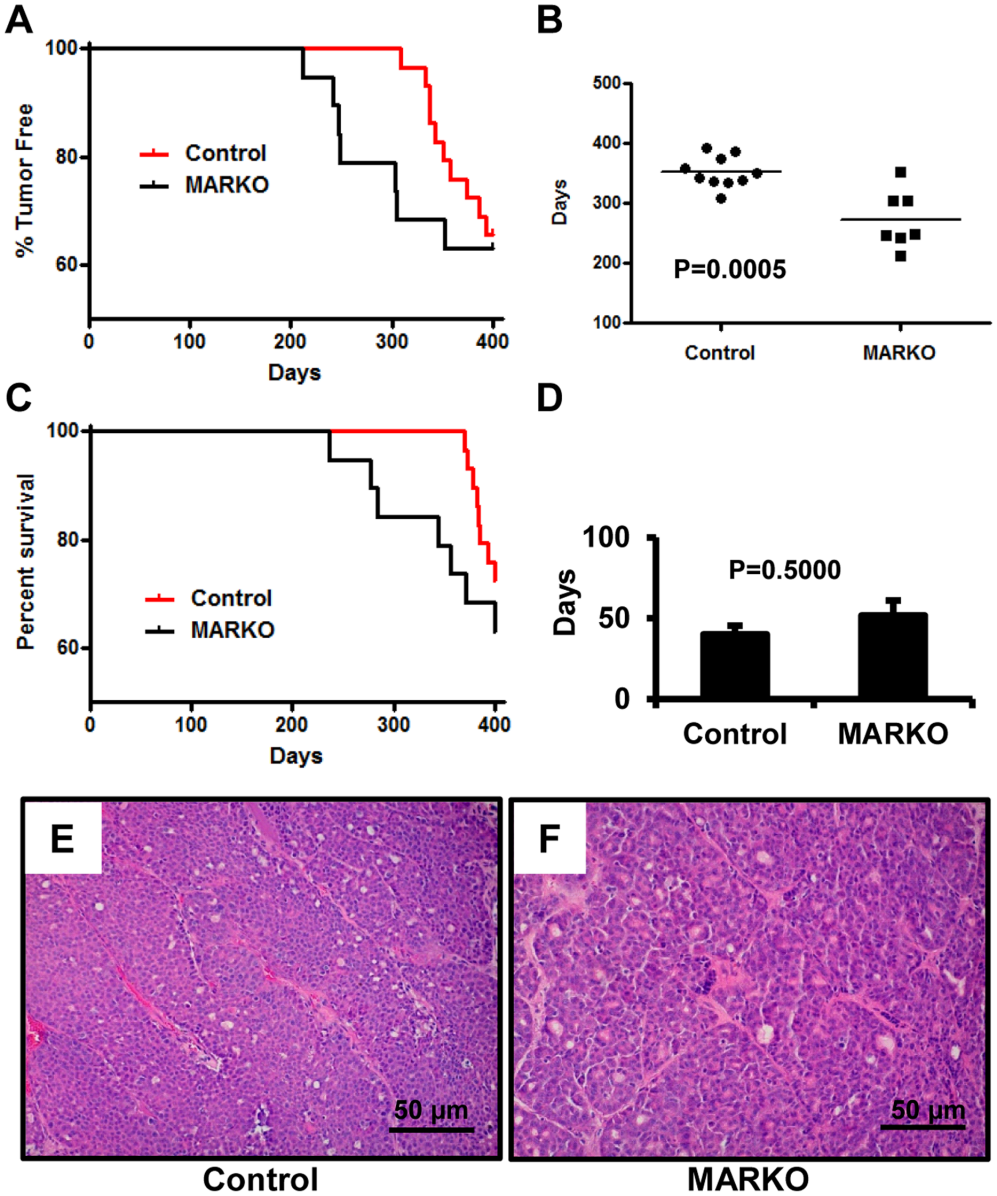
MARKO mice show an earlier onset of breast tumors in MMTV-NeuNT mice. A. Mice were examined two to three times per week starting at 6 months for the presence of palpable tumors. Age of incidence was recorded as the first day at which a palpable tumor was detected. Percentage of tumor free mice was plotted versus the age of the mice in days. Hazard ratio = 0.2340 and 95% CI ratio of = 0.06054 to 0.9047. B. Mean age of tumor incidence was calculated for tumor bearing MARKO (273+/−18.36 days, n = 7) and control (352.5+/−8.263 days, n = 10) mice, p = 0.0005. C. Survival of MARKO and Control MMTV-NeuNT females. Percentage of surviving mice was plotted versus age (p = 0.3499 at 400 days). Hazard ratio = 0.6023 and 95% CI of ratio = 0.2080 to 1.744. D. Average time in days between tumor detection and sacrifice, due to tumor burden prior to 400 days of age, p = 0.5000. Representative hematoxylin and eosin staining of Control (E) and MARKO (F) tumors. Scale bar = 50 µm.

The first tumors in MARKO mice were observed almost 100 days prior to the appearance of palpable tumors in control mice (Figure 3A). AR deficiency in the mammary glands of female mice dramatically accelerated the onset of MMTV-NeuNT driven tumors. In female mice that developed tumors within 400 days, MARKO mice had a significantly lower average age of tumor onset (273±18.36 days) compared to their control littermates (352.5±8.263 days, P = 0.0005) (Figure 3B). To evaluate whether loss of AR affected survival, females were sacrificed when tumors grew to approximately 1.5 cm in diameter, an animal had multiple tumors, or showed signs of morbidity, and the survival curves were calculated. The similar average size of tumors in the two groups (Control = 1.91±0.27 g and MARKO = 1.71±0.47 g) demonstrated that the mice were sacrificed without preferential selection. Concordantly with an earlier onset of tumors, the survival rate in MARKO females within the first 375 days was significantly reduced (P = 0.0208, Log-rank Test). However, no significant difference was observed in the survival rate between control and MARKO mice during the full course of this study (400 days) (Figure 3C, P = 0.2376). Similar numbers of females in both groups (36.84% (7/19) in MARKO versus 27.58% (8/29) in Control) developed lethal cancer within the period of the study. The time between tumor detection and euthanasia between two groups was slightly lower in the Control group, however the difference was not statistically significant (Figure 3D). At 400 days, 2 female control mice were determined to have small mammary tumors, while no MARKO mice had detectable tumors. Therefore at the completion of the study, 58.6% (17/29) of control mice and 63.16% (12/19) of MARKO females were tumor free. Similar numbers of tumor-bearing mice in the two groups had multiple tumors (41.6%, 5/12 in Control and 43.8%, 3/7 in MARKO). Although MARKO females displayed an earlier onset of MMTV-NeuNT tumors, the cell proliferation index evaluated by Ki67 staining in tumors isolated at sacrifice was similar in both groups (Fig. S1). Histological analysis of tumors showed that tumors were poorly differentiated and had a uniform morphology comprised of compact adenocarcinoma cells, characteristic for this model [7] with limited portions of intratumoral stroma (Figure 3E and F). Histological grading of the tumors was similar between both groups (Table 1). No metastases were detected in our experiments.

**Table 1:** Histopathological tumor grading in Control and MARKO mice.

Mouse #	Genotype	Necrosis	Mitosis per 40X (field diameter 0.44 mm)	Grade
**C424**	Control	Extensive	8	High
**C589**	Control	Extensive	5	High
**C745**	Control	None	2	Low
**C951**	Control	None	1	Low
**C756**	Control	Focal	2	Low
**C764**	Control	None	2	Low
**C779**	Control	None	1	Low
**C786**	Control	Focal	1	Low
**C410**	Control	Tumor was too small and was used up for RNA analysis		
**C299**	MARKO	None	1	Low
**C472**	MARKO	Focal	5	High
**C582**	MARKO	None	1	Low
**C658**	MARKO	Focal	4	High
**C775**	MARKO	Focal	3	Low
**C777**	MARKO	Focal	1	Low
**C755**	MARKO	Focal	4	High
**C754**	MARKO	Tumor was too small and was used up for RNA analysis		

Mitotic count and presence/absence of necrosis were used as grading criteria in this study. Tumors with a low mitotic count (<4 hpf) and focal (<5%) or no necrosis correspond to low grade carcinoma while those with a high mitotic rate (≥4/5 hpf) and necrosis (>5%) are considered high grade. Low and high grade carcinomas were detected in both Control and MARKO groups.

Previously it was shown that MMTV-NeuNT caused the development of mammary tumors in male mice [7]. In our study only 10.9% of males developed tumors within 450 days, including 2/33 in the MARKO group (∼6%) and 5/31 in the control group (∼16%), and of these mice only 3 were sacrificed due to tumor burden. PCR analysis showed the presence of deleted *Ar^Δ^* allele in all samples of genomic DNA isolated from MARKO male mammary glands (Figure 1B). The low number of tumor-bearing mice during the course of this study prevented further evaluation of the role of AR deletion in MMTV-NeuNT male mice.

### AR does not Suppress Mammary Gland Mitogenic Signaling Pathways

**Figure 4:**
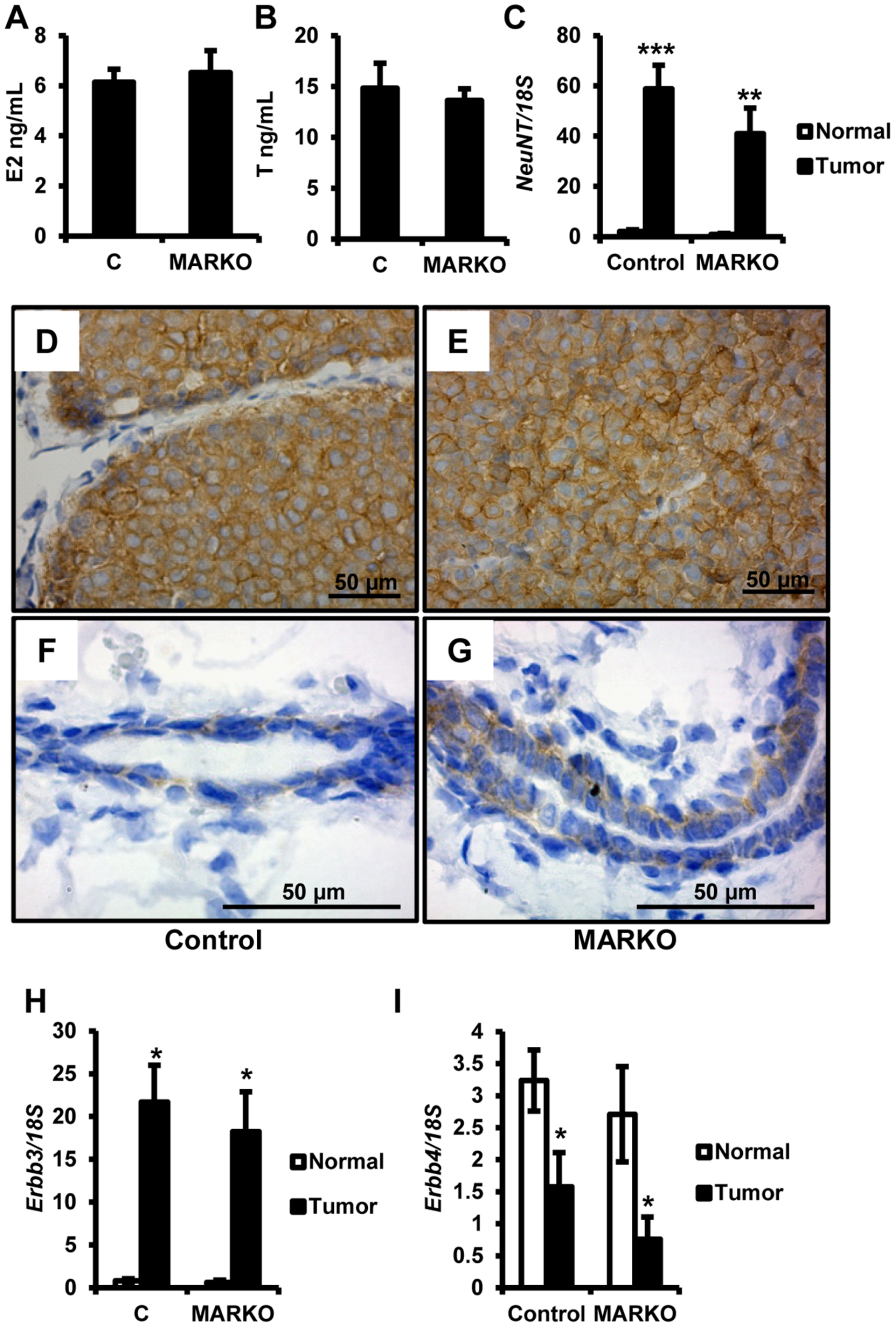
Elevated ERBB2 and ERBB3 expression in Female MMTV-NeuNT tumors. A. Serum obtained at sacrifice from control (n = 11) and MARKO (n = 10) mice was analyzed for estradiol levels (E2). B. Serum from A was analyzed for testosterone (T) levels. C. MMTV-NeuNT expression. At the time of sacrifice, tumors were harvested and non-tumor bearing mammary glands were dissociated from the fat pad and RNA was prepared as described in the methods section. *NeuNT* expression was analyzed by quantitative RT-PCR and normalized to 18S expression. Expression of *NeuNT* in tumors (Control n = 9 and MARKO n = 7) for MARKO and Control mice and normal mammary glands (Control n = 26, MARKO = 15) was normalized to expression in non-tumor bearing mammary glands of the Control group. D-G. Immunohistochemical detection of ERBB2 in tumors, Control (D) and MARKO (E) at 40X magnification, and in normal mammary ducts, Control (F) and MARKO (G) at 100X magnification. Gene expression analysis of *Erbb3* (H) and *Erbb4* (I) in the same samples as in C. *denotes P<0.05, **P<0.01 and ***P<0.005.

To determine if AR loss influenced hormone status in our model we evaluated the serum concentrations of estradiol and testosterone. No changes in serum estradiol or testosterone levels were detected between control and MARKO mice (Figure 4A and 4B). Expression of *Esr1*, *Pr* and downstream target genes of both receptors: amphiregulin (*Areg*), receptor activator of nuclear factor kappa-B ligand (*Rankl*), and wingless-type MMTV integration site family, member 4 (*Wnt4*) were also unchanged in non-tumor bearing mammary glands of MARKO mice compared to controls (Fig. S2).

In both MARKO and control mice, *Errb2* transgene expression was dramatically increased in tumors compared to normal non-tumor bearing mammary glands from the same animals (Figure 4C). Elevated *Erbb2* expression was similar in both MARKO and control tumors (p = 0.2197). Increased ERBB2 protein levels were detected in tumors (Figure 4D and 4E compared to 4F and 4G), corresponding to the elevated mRNA levels.

Overexpression of ERBB2 in human patients is associated with elevated expression or activity of ERBB3 [22]. Elevated levels of ERBB3 protein have been noted in tumors of ERBB2 transgenic mice [10]. We compared the expression of *Erbb3* in tumors from control and MARKO mice to non-tumor bearing mammary glands. Strikingly, increased expression of ERBB2 was associated with 18 to 20 fold increased expression of endogenous *Erbb3* (Figure 4H). Loss of *Ar* in MARKO mice did not alter increased *Erbb3* expression. To evaluate whether *Erbb3* upregulation in MMTV-NeuNT tumors was selective or whether these tumors displayed elevated expression of other ERBB family members, we also examined the expression of *Erbb4*. Expression of *Erbb4* in both normal cohorts was similar and was significantly reduced in tumors (Figure 4I) (normal versus tumor in Control p = 0.0310 and MARKO p = 0.0272). No significant difference was noted between tumors from the two groups. This suggests that *Erbb3* is selectively upregulated to cooperate with ERBB2 driven tumorigenesis.

### ERα and PR Signaling is Maintained during AR Loss in Ductal Epithelium

**Figure 5:**
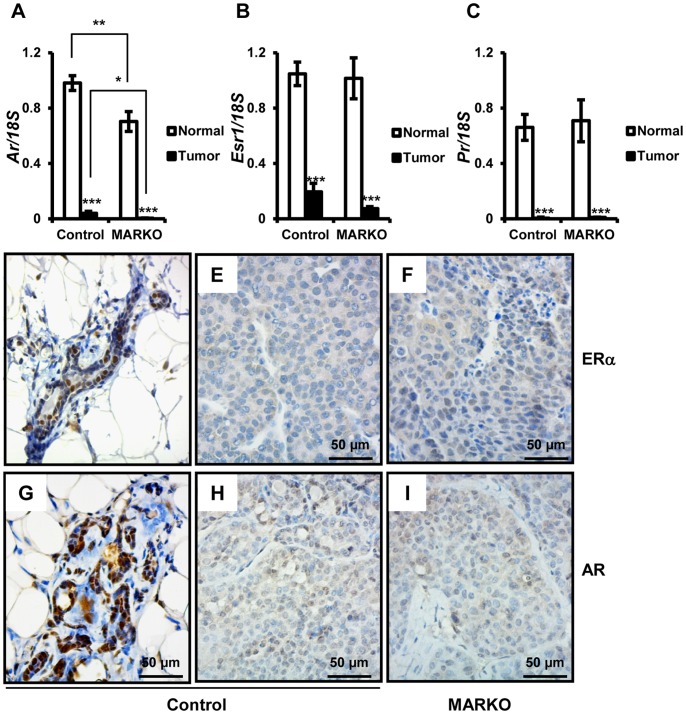
Steroid receptor expression is reduced in MMTV-NeuNT tumors. At the time of sacrifice, tumors were harvested and non-tumor bearing mammary glands were dissociated from the fat pad. RNA was prepared as described in the methods section. Levels of *Ar* (A), *Esr1* (B), *Pr* (C) and 18S were analyzed by quantitative RT-PCR (Control normal = 26, Control tumor = 9, and MARKO normal = 15 and MARKO tumor = 7). Expression of each receptor was normalized to 18S. Receptors in tumors for MARKO and Control mice and normal MARKO glands were normalized to expression in non-tumor bearing mammary glands from the Control group. *denotes P<0.05, **P<0.01 and ***P<0.005. Immunohistochemical detection of ERα in the normal mammary gland (D) of Control mice and tumors from Control (E) and MARKO (F) mice. Immunohistochemical detection of AR in the normal mammary gland of Control mice (G) and tumors from Control (H) and MARKO (I) mice. Scale bar = 50 µm.

Hormonal independence of breast cancer can occur with disease progression and induction of ERBB2 signaling [33], [34]. Therefore we evaluated the expression of sex steroid receptors in tumors obtained from control and MARKO mice and compared them to each other and to non-tumor bearing mammary glands. *Ar* expression was significantly reduced in MARKO tumors compared to tumors from control mice (Figure 5A). In addition, regardless of genotype, we observed significant loss of *Ar* expression in MMTV-NeuNT tumors compared to normal mammary glands (Figure 5A).

Similarly to *Ar*, *Esr1* and *Pr* expression was significantly reduced in MMTV-NeuNT tumors compared to non-tumor bearing mammary glands (Figure 5B and C). We observed similar *Esr1* and *Pr* expression in MARKO tumors and tumors obtained from control mice (*Esr1* p = 0.1020 and *Pr* p = 0.4385) (Figure 5B and C). Correlating with reduced mRNA levels of steroid receptors in tumors, we also observed reduced protein staining for ERα (Figure 5D–F) and AR (Figure 5G–I) in MMTV-NeuNT tumors.

**Figure 6:**
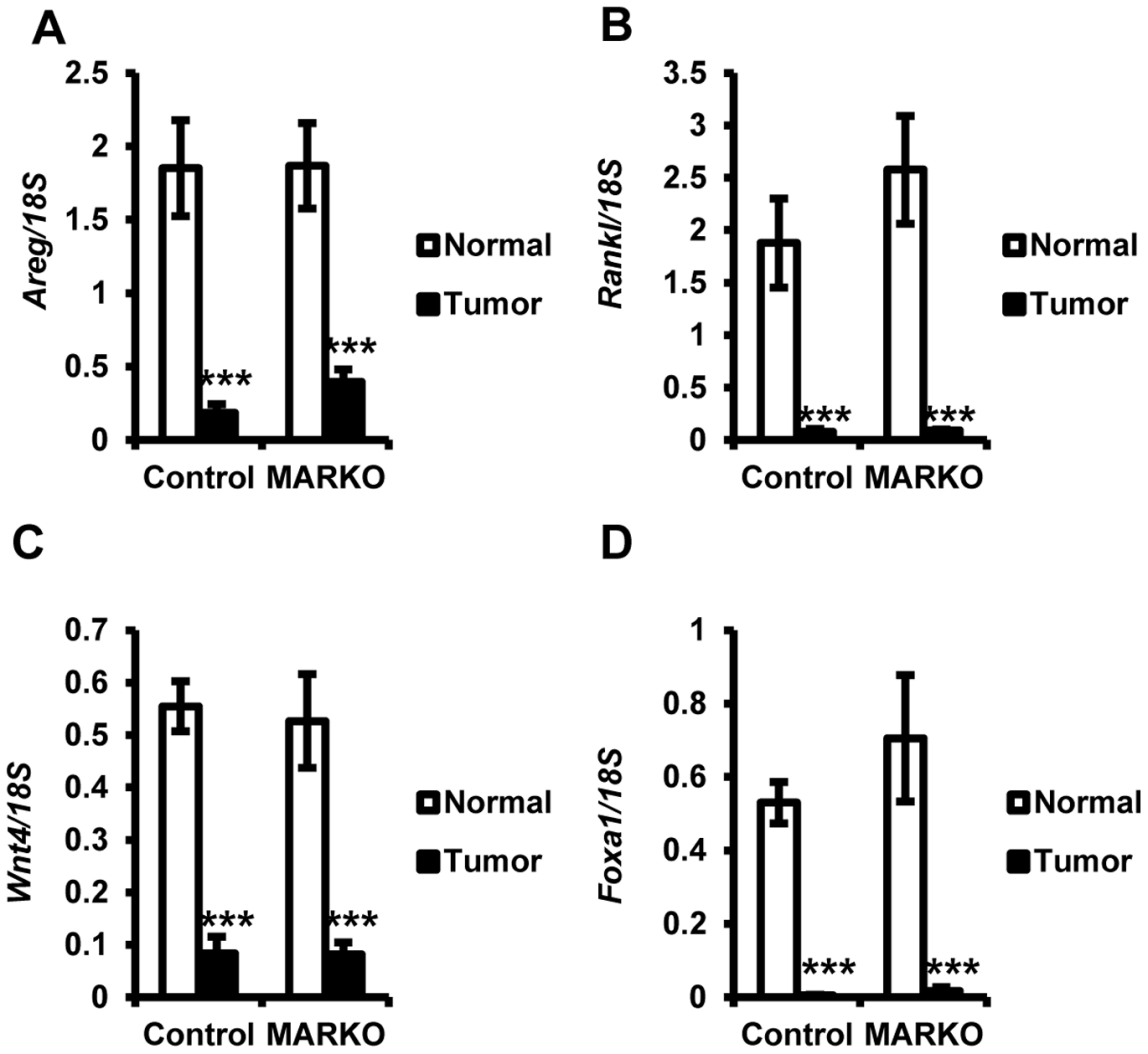
Downstream targets of steroid receptors are reduced in MMTV-NeuNT tumors. Tumors and non-tumor bearing mammary glands were evaluated for the expression of ERα and PR downstream target genes. Expression of each gene was normalized to 18S. Gene expression in tumors for MARKO and Control mice and normal MARKO glands were normalized to expression in non-tumor bearing control mammary glands (Control normal = 26, Control Tumor = 9, MARKO normal = 15 and MARKO tumor = 7). Reduced expression was seen in tumors compared to normal tissue for *Areg* (A), *Rankl* (B), *Wnt4* (C) and *Foxa1* (D). ***P<0.005, for normal versus tumor expression.

To confirm reduced steroid receptor signaling in MMTV-NeuNT tumors, the expression of known ERα and PR target genes (*Areg, RankL and Wnt4*) were examined. As was observed for the receptors, expression of these target genes was significantly reduced in MMTV-NeuNT tumors compared to normal mammary glands (Figure 6). Expression of *RankL* was elevated in normal mammary glands of MARKO mice compared to Controls, although it failed to reach statistical significance (p = 0.3036). *Pr* is an ERα target gene and therefore the reduced expression of *Pr* and downstream genes may be the consequence of reduced ERα expression. This data further confirms that steroid receptor signaling is reduced in MMTV-NeuNT tumors. No significant difference in expression of *Areg*, *RankL,* or *Wnt4* was detected between the tumors of control and MARKO mice (Figure 6). This was consistent with similar levels of *Esr1* and *Pr* expression in tumors.

Since the steroid receptors themselves were diminished in MMTV-NeuNT tumors, we examined the status of FOXA1, a pioneer factor closely associated with ERα and AR function [35], [36] and with ERBB2 expression [37], [38]. Interestingly, we observed a significant loss of *Foxa1* expression in tumors (Figure 6D).

### MMTV-NeuNT Expression does not Affect Steroid Receptor Status Prior to Tumor Formation

**Figure 7:**
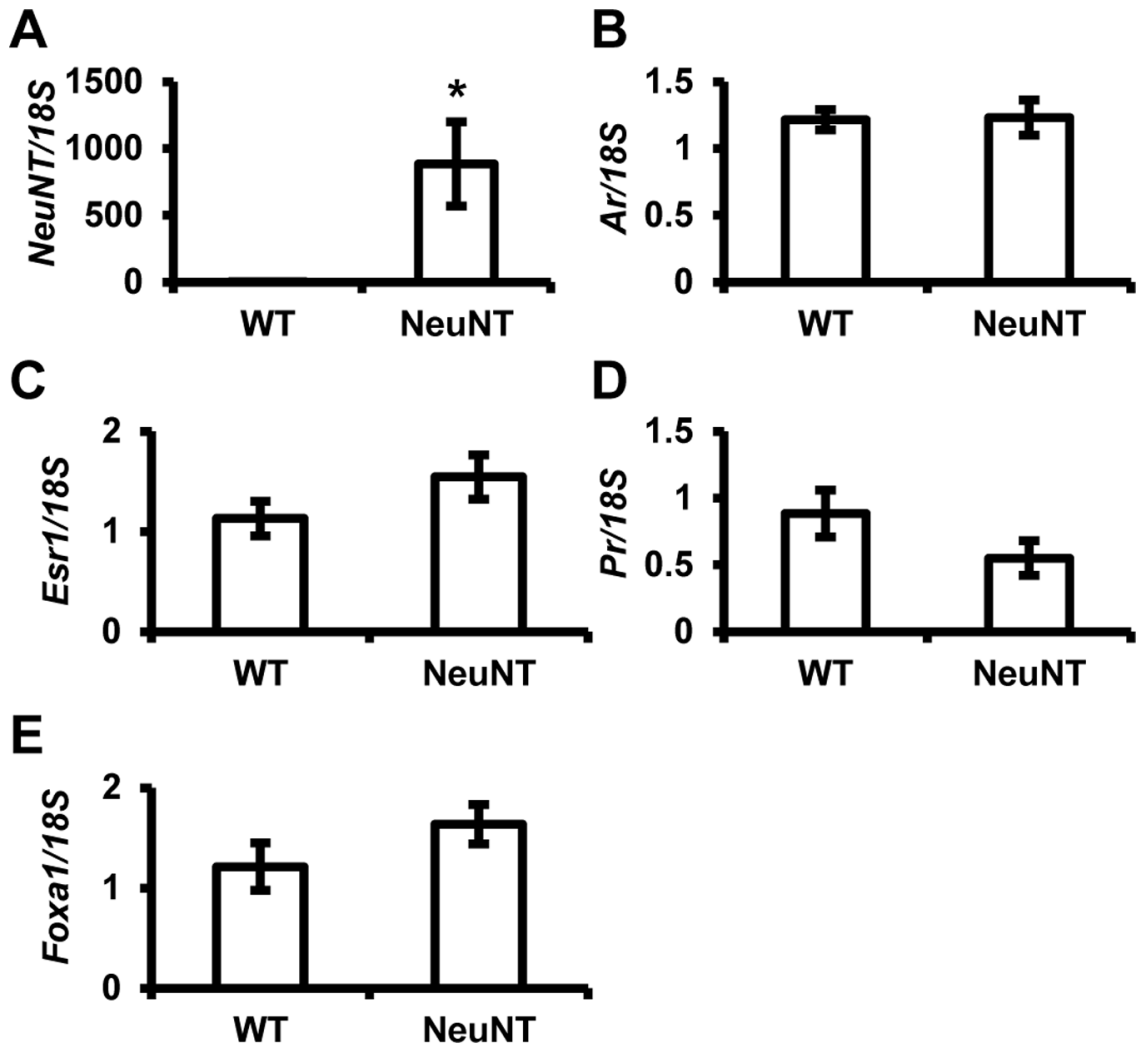
MMTV-NeuNT does not affect steroid receptor expression in normal mammary glands. MMTV-NeuNT expressing (NeuNT) and non-expressing (WT) mammary glands were dissociated from the fat pad. RNA was prepared as described in the materials and methods section. Expression of *NeuNT* (A), *Ar* (B), *Esr1* (C), *Pr* (D), and *Foxa1* (E) were analyzed by qRT-PCR (WT n = 8 and MMTV-NeuNT n = 10). Expression of each gene was normalized to 18S. (*denotes P<0.05).

Loss of steroid receptor and *Foxa1* expression in MMTV-NeuNT tumors indicated that *Erbb2* expression drove the loss of these markers and that their loss may be associated with tumor etiology. To evaluate whether *Erbb2* altered the expression of these genes prior to tumor formation, we compared their expression in normal mammary glands from MMTV-NeuNT expressing mice and nontransgenic mice with the same mixed genetic background (minus MMTV-NeuNT). Expression of the rat *Erbb2* transgene was detected only in MMTV-NeuNT mice (Figure 7A). No significant change in *Ar*, *Esr1,* or *Pr* expression was observed (Figure 7B, 7C, and 7D). *Foxa1* showed no change in expression between MMTV-NeuNT expressing and non-expressing mammary glands (Figure 7E).

## Discussion

To investigate the role of AR in ERBB2 driven tumorigenesis we created a mouse model that expressed an activated mutant of rat ERBB2 [7] and were heterozygous for *Ar* deletion. Due to X chromosomal inactivation in females the floxed or wild type *Ar* allele may be inactivated. In luminal epithelial cells of MARKO mice where the floxed allele is inactivated, Cre recombinase will fail to ablate AR and therefore these mice will retain a portion of cells with normal AR expression. This was reflected in AR expression analysis of MARKO mammary glands, which displayed an approximately 30% decrease in *Ar* at the mRNA level. We utilized only partial dissociation of the mammary gland to evaluate gene expression. Therefore, the presence of stromal cells, vasculature, and some fat cells likely reduced the true extent of *Ar* deletion. Possible proliferative advantage of one genotype over the other and penetrance of MMTV-cre expression may also contribute to varied recombination frequency. AR histological staining confirmed that MARKO mice display a significant reduction in the number of luminal epithelial cells positive for AR.

The current model does not generate complete ablation of *Ar* across the entire mammary gland; rather it creates a mixed population of epithelial cells expressing normal levels of AR and cells with complete lack of expression. Genetic alterations that give rise to breast cancer or promote tumor progression are thought to occur within a limited number of cells. Thus, MMTV-cre, MMTV-NeuNT, *Ar^fl^/+* mice may be a valuable *in vivo* model for ARs role in breast carcinogenesis.

In the current study, we noticed a considerable delay in the onset of tumors in control mice (MMTV-NeuNT, Ar*^fl^/+*) compared with the incidence reported for the original parent colony ((FVB-Tg(MMTV-ErbB2)NK1Mul/J), which reported 50% incidence in female mice by 25 weeks of age [7]. This disparity is possibly due to differences in the genetic background of mouse strains; the original line is on an FVB background, whereas *Ar^fl/fl^* mice are on a C57BL/6 background. C57BL/6 mice are reported to be more resistant to multiple types of tumors [39], [40], [41]. Furthermore, we observed almost no tumor development in our male cohort of mice over a period of 65 weeks. We were interested to determine if AR deletion also affected the susceptibility of male breast cancers, since men with partial or complete androgen insensitivity (AIS) have an elevated risk of breast cancer. AIS associated breast cancer risk is typically associated with elevated estrogen exposure [42]. The lack of tumor development in male mice was also probably due to the mixed background utilized in the current study.

It has previously been reported that global AR knockout increases the susceptibility of female mice to DMBA-induced mammary tumors [26]. Increased tumor susceptibility in ARKO females was associated with elevated proliferation of luminal epithelial cells in virgin mice. Increased proliferation in ARKO females was likely due to the loss of AR antagonism of ERα signaling. In the ARKO study, mammary glands were selectively collected at 8 weeks of age during the estrus phase of the estrous cycle, at which time estrogens exert their largest influence. In the present study, normal mammary glands were collected either at sacrifice due to tumor burden or once females reached 400 days of age independently of the estrous cycle. While we did not observe significant change in proliferation rates, the possibility that MARKO mice have elevated proliferation in the AR depleted luminal epithelium during estrus cannot be excluded.

Inhibition of AR signaling by the antagonist flutamide in postpubertal mice significantly increased proliferation of mammary epithelial cells [25], indicating that the blockade of AR signaling disrupts the homeostatic balance between estrogen and androgen signaling thus leading to unconstrained estrogen driven proliferation. As seen in the current study, suppression of AR function did not influence estradiol levels or ERα expression. Thus, it is the loss of AR signaling in the normal mammary gland under the influence of normal hormone levels and female steroid receptor status that increases the susceptibility of the mammary gland to further tumor-initiating insults.

Elevated estrogen exposure is a risk factor for breast cancer development and continuous exposure to estrogen in rodents is sufficient to drive the formation of mammary tumors [43]. ERα knockout (ERKO) inhibits the development of mammary tumors in mice [18], [44]. In the MMTV-NeuNT mouse model both ERα and NeuNT are required for tumorigenesis as ERKO mice display significant delay in the development of MMTV-NeuNT tumors. The AR antagonizes the mitogenic activity of the estrogen receptor [45] suggesting that AR may oppose ERα activity during the early stages of MMTV-NeuNT driven tumorigenesis and AR loss leads to the accelerated onset of mammary tumors.

Distinct from human breast cancers, which frequently retain steroid receptors, mouse models of breast cancer frequently lack steroid receptors and develop hormone independence [46], [47], [48]. In the present study we demonstrate that in the MMTV-NeuNT mouse model of breast cancer the expression of AR in these tumors is reduced. This suggests that although the AR suppresses the formation of ERBB2 driven tumors, once these tumors are established they may become less reliant or responsive to androgens or ovarian hormones.

The function of ERα and AR are dependent on the pioneer factor FOXA1 and mammary glands fail to develop in FOXA1 knockout animals [49], [50]. Although the precise significance of FOXA1 expression in breast cancer is unclear, FOXA1 expression has been shown as a positive prognostic factor in ERα positive tumors. A strong correlation between FOXA1 overexpression and ERBB2 in ERα negative tumors has also been identified [51], [52]. There are reports of crosstalk between FOXA1, AR and ERBB2 [52], [53] and of mutual regulation of expression by FOXA1 and ERα [49]. In the present study we demonstrate that MMTV-NeuNT tumors have reduced expression of *Foxa1* relative to non-tumor bearing mammary glands. It would be interesting to determine if loss of FOXA1 drives the development of hormone independent tumors through perturbed steroid receptor signaling or if *Foxa1* expression is diminished due to the reduced expression of ERα and/or AR.

ERBB3 overexpression and activation has been detected in tumors from several lines of ERBB2 transgenic mice [10]. However, unlike the previous report that detected minimal elevation in *Erbb3* transcription, we note a significant up regulation of *Erbb3* mRNA in MMTV-NeuNT tumors. This indicates that in different mouse models of ERBB2 oncogenic activation endogenous ERBB3 is selectively upregulated, although by different mechanisms. Although mutated ERBB2 is capable of homodimerizing, ERBB2/ERBB3 heterodimers can utilize mouse growth hormone signaling and may be required for tumorigenesis. We also observed no increase in *Erbb4* expression. This suggests that NeuNT transformation selectively amplifies *Erbb3* expression to drive tumorigenesis.

Diminished expression of AR in the mammary glands of female mice leads to dramatically earlier onset of MMTV-NeuNT induced mammary tumors. Interestingly, AR loss did not result in an increase in the percentage of tumor-bearing mice in the MARKO group compared to controls. Female ARKO mice do not develop mammary tumors [26] and, in our study, neither do female MARKO mice that do not have the MMTV-NeuNT transgene. Thus, AR inactivation alone does not predispose the mammary gland to mammary tumors. This indicates that the AR opposes mammary gland transformation and its loss predisposes mammary epithelial cells to malignant growth instigated by other oncogenes.

MARKO tumors display the same profile as control tumors; proliferation rate, tumor growth rate, steroid receptor and downstream signaling profiles. In addition, *Foxa1* and *Erbb3/Erbb4* expression levels are similar. Therefore it is the loss of AR function that makes it easier for epithelial cells expressing ERBB2 to undergo malignant transformation.

## Supporting Information

Figure S1 Tumor cell proliferation is not influenced by AR status. Representative pictures of Control (A) (n = 12) and MARKO (B) (n = 8) tumors stained for Ki67. (C) Staining in A and B were quantified and are shown as the percentage of Ki67 positive cells. Scale bar = 50 µm.
(TIF)

Figure S2 Steroid receptor signaling is intact in normal MMTV-NeuNT expressing mammary glands. MMTV-NeuNT expressing non-tumor bearing mammary glands from Control (n = 24) and MARKO (n = 15) mice were dissociated from the fat pad. RNA was analyzed for the expression of *Er*α (A), *Pr* (B), *Areg* (C), *RankL* (D), *Wnt4* (E). Expression of each gene was normalized to 18S.
(TIF)

Table S1 Sequences of the forward and reverse primers used for the corresponding gene expression analysis by qPCR.
(XLSX)
