# Supplementary figures and images for: Reduced Androgen Receptor Expression Accelerates the Onset of ERBB2 Induced Breast Tumors in Female Mice
Source: PLoS One. 2013 Apr 8;8(4):e60455. doi: 10.1371/journal.pone.0060455 (PMC3620158; doi:10.1371/journal.pone.0060455)

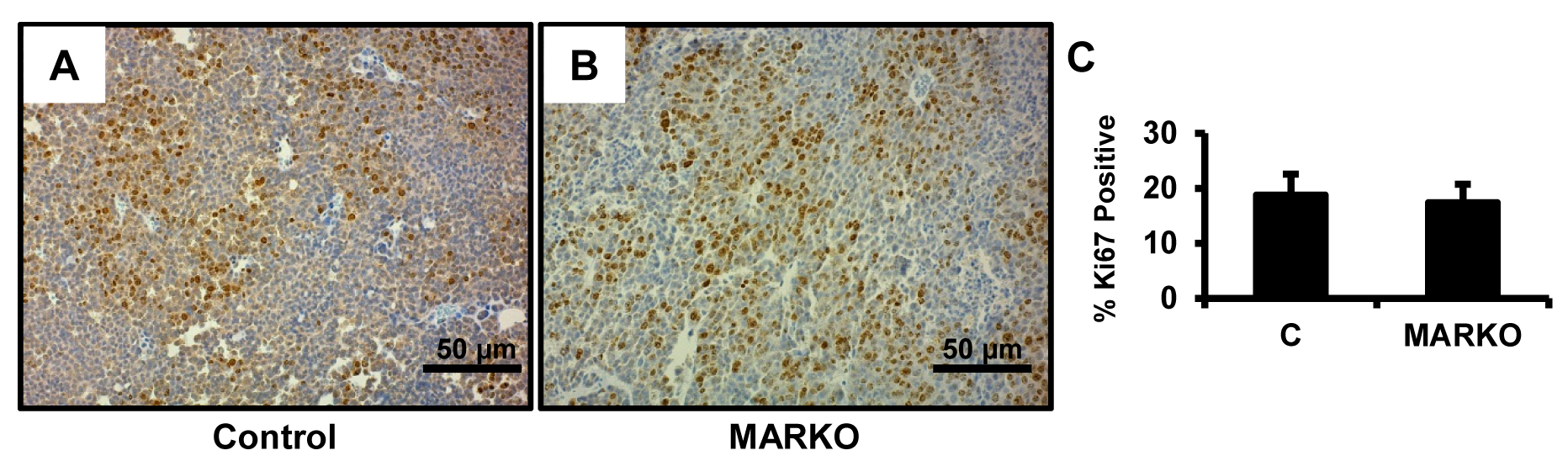

Supplement: Figure S1 — Tumor cell proliferation is not influenced by AR status. Representative pictures of Control (A) (n = 12) and MARKO (B) (n = 8) tumors stained for Ki67. (C) Staining in A and B were quantified and are shown as the percentage of Ki67 positive cells. Scale bar = 50 µm. (TIF) [file pone.0060455.s001.tif]

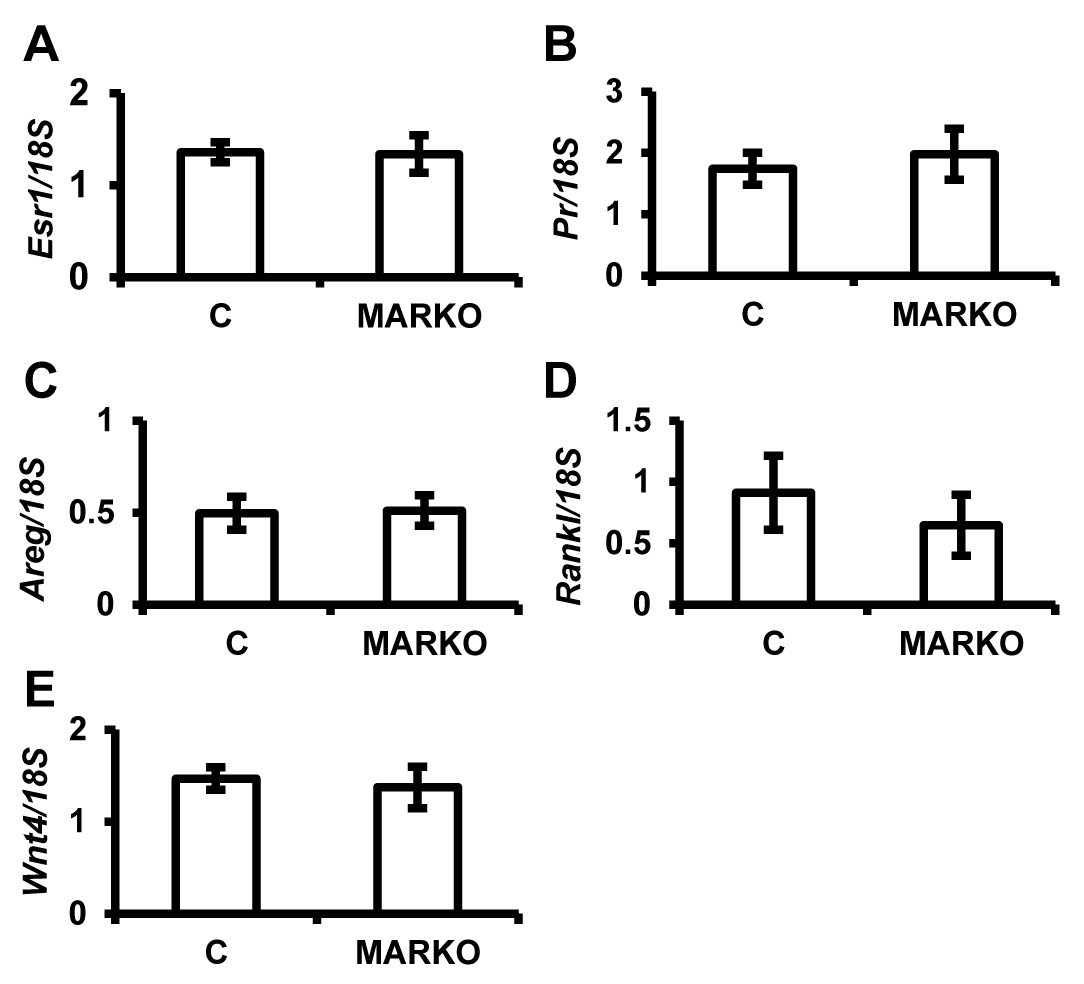

Supplement: Figure S2 — Steroid receptor signaling is intact in normal MMTV-NeuNT expressing mammary glands. MMTV-NeuNT expressing non-tumor bearing mammary glands from Control (n = 24) and MARKO (n = 15) mice were dissociated from the fat pad. RNA was analyzed for the expression of Erα (A), Pr (B), Areg (C), RankL (D), Wnt4 (E). Expression of each gene was normalized to 18S. (TIF) [file pone.0060455.s002.tif]
